# Risk factors associated with preferential lateral lymph node metastasis in papillary thyroid carcinoma

**DOI:** 10.1002/cam4.6567

**Published:** 2023-10-31

**Authors:** Liang Shao, Zhihong Wang, Wenwu Dong, Wei Sun, Hao Zhang

**Affiliations:** ^1^ Department of Thyroid Surgery The First Hospital of China Medical University Shenyang Liaoning Province P. R. China

**Keywords:** central lymph node, lateral lymph node, metastasis, papillary thyroid cancer, risk factor

## Abstract

**Background:**

Lateral lymph node metastasis (LLNM) is common in patients with papillary thyroid cancer (PTC), usually occurring after central lymph node metastasis (CLNM). However, some patients experience LLNM without first developing CLNM. This study aimed to identify the risk factors for developing LLNM without CLNM.

**Methods:**

We retrospectively reviewed 421 patients diagnosed with PTC who underwent lobectomy or total thyroidectomy with central and ipsilateral lateral lymph node dissection. We collected clinicopathological data and used univariate and multivariate logistic regression analyses to determine the risk factors associated with LLNM without CLNM.

**Results:**

The LLNM without CLNM frequency was 18.3% (77/421). Univariate analyses demonstrated that age over 55 years, primary tumor in the upper portion of the thyroid, the number of central lymph node (CLN) and LLNM, primary tumor size, and the summed size of multi‐foci tumors smaller than 1 cm were significantly associated with LLNM without CLNM (*p* < 0.05). Multivariate analysis revealed that LLNM without CLNM was more likely to occur in patients aged ≥55 years (odds ratio [OR], 2.309; 95% confidence interval [CI], 1.133–4.704; *p* = 0.021), and primary tumor in the upper portion of the thyroid (OR, 0.524; 95% CI, 0.295–0.934; *p* = 0.028).

**Conclusion:**

The lymph node metastasis pattern in patients with PTC is not constant. Therefore, surgeons should evaluate the lateral lymph nodes, especially in patients older than 55 years or when the primary tumor is in the upper portion of the thyroid.

## 1 INTRODUCTION

Over the past several decades, thyroid cancer has become the most common endocrine malignancy.^1^ Papillary thyroid cancer (PTC) is the main histological type, accounting for over 90% of all thyroid cancers. Previous studies on patients with PTC have reported 10‐ and 15‐year survival rates of >91% and >87%, respectively. Therefore, PTC has been considered as low‐risk cancer.^2^, ^3^ Although regional lymph node metastasis (LNM) is found in approximately 30%–80% of patients with PTC, numerous studies have reported that LNM does not affect their prognosis.^4^ However, some surgeons have reported that lateral lymph node metastasis (LLNM) was associated with distant metastases and cancer‐related death in patients with PTC.^5^, ^6^ Reoperation associated with cancer recurrence in the lymph nodes or thyroid region will increase costs and the risk of complications, and, in some instances, affect the patient's psychological response. Consequently, these factors are likely to affect the patient's quality of life. The American Thyroid Association (ATA) recommended that surgeons should not dissect the central and lateral lymph nodes (LLNs) unless there is definite evidence of metastasis such as biopsy‐proven PTC.^7^ Therefore, it is vital to evaluate for LNM before surgery, especially in patients whose ultrasonography results did not indicate any signs of LNM.

LLNM typically occurs after central lymph node metastasis (CLNM), a sequence attributed to the lymphatic drainage system. Some PTC metastasize to the LLNs without CLNM, a condition referred to as skip metastasis.^8^, ^9^ However, we suggest that the name is inappropriate because there has been no evidence to prove that the thyroid lymphatic drainage system first passes through the central lymph nodes (CLNs). Therefore, we suggest that the most appropriate name for this condition should be LLNM without CLNM. Additionally, the evaluation of LLNM without CLNM is less predictable because the CLNM are absent.

This study analyzed clinicopathological data of patients with LLNM with and without CLNM to estimate the risk factors for LLNM without CLNM. The findings reported in this study could help surgeons evaluating the LLNs in patients with PTC before surgery.

## 2 METHODS

The Institutional Review Board of The First Hospital of China Medical University approved this study, and informed consent was not required due to the retrospective nature of this research. We reviewed the medical records of 421 patients with PTC who underwent lobectomy or total thyroidectomy with central and ipsilateral LLN dissection (LLND) in our hospital between January 2013 and December 2016. All patients were diagnosed with PTC with LLNM by a pathologist with at least 10 years of experience working at the pathology center in our hospital. Patients who met any of the following were excluded: not the first surgery, had other neck or head carcinoma, history of neck radiation exposure, other types of thyroid malignancy or with high‐risk cell types of PTC, and individuals whose complete case data were not available.

A general physical examination was performed to all patients before surgery. Ultrasonography and enhanced computed tomography (CT) were performed to evaluate the characteristics of the thyroid gland, including the location and diameter of the tumors and CLN or LLN assessment. The imaging results were evaluated by at least two experienced radiologists and surgeons. LLND was performed after lobectomy or total thyroidectomy with ipsilateral CLN dissection (CLND) in instances where the preoperative evaluation revealed the presence of LLNM. The LLNs were then divided into four levels (II–V) according to the criteria recommended by the American Head and Neck Society^10^ and the ATA.^7^ Clinical characteristics, including sex, age, tumor size, total tumor size, tumor multifocality, extrathyroidal extension (ETE), primary tumor location, presence of goiter nodules, Hashimoto's thyroiditis (HT), CLNM, and LLNM were determined based on the preoperative examination, intraoperative findings, and postoperative pathological reports. Total tumor size was calculated as the sum of all tumor diameters when more than one tumor was present. The patients were divided into two groups according to the presence of CLNM.

The tumor location was divided into the upper, middle, and lower portions and full gland based on the pre‐operation ultrasonographic results and intraoperative findings recorded by the surgeons. The CLNs and LLNs were removed and divided into the corresponding levels. A pathologist then evaluated them for the presence of metastasis. Moreover, we analyzed the relationship between LLNM and the tumor location. In cases with multifocal tumors, we only enrolled patients with one tumor ipsilateral to the LLNM.

Data management and statistical analyses were performed using IBM SPSS Statistics for Windows, Version 21.0 (IBM Corp.). Continuous and categorical data are expressed as mean ± standard deviation (SD) and rates, respectively. Mann–Whitney *U*‐test and chi‐squared or Fisher's exact test (two‐tailed) compared the groups for continuous and categorical data, respectively. Univariate analyses were performed using the chi‐squared test or Fisher's exact test. Factors with *p* < 0.05 were further analyzed by multivariate analyses, using binary logistic regression. Data assessing the role of potential risk factors are presented as odds ratios (ORs) with 95% confidence intervals (CIs).

## 3 RESULTS

**FIGURE 1:**
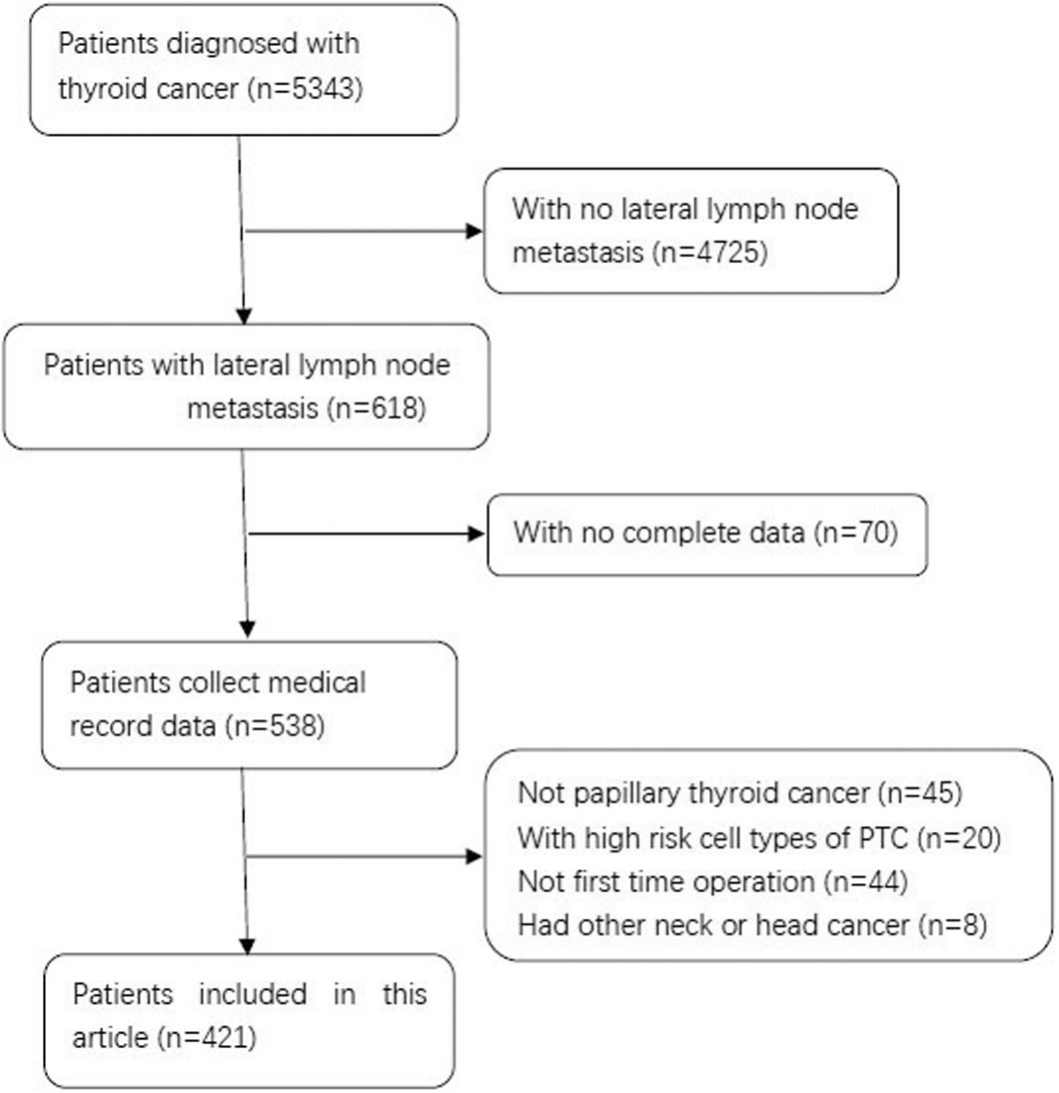
Flowchart showing the patient selection process in this study.

We have evaluated the data of 5343 patients diagnosed with thyroid cancer in our hospital and excluded 4725 of them for not having LLNM. Another 70 patients were excluded for incomplete data. After excluding patients for the other exclusion criteria, our study included 421 patients (Figure 1).

Of the included patients, 124 were males, and 297 were females, with a ratio of 1:2.4. The mean age was 40.5 ± 12.1 (range, 18–78) years. 363 patients (86.2%) were under 55 years old, 236 (56.1%) had nodular goiter, and 119 (28.3%) had HT confirmed by postoperative pathology.

The mean primary tumor and total tumors sizes were 2.0 ± 1.1 and 2.2 ± 1.3 cm, respectively. Microcarcinoma (≤1.0 cm) was found in 114 patients (27.0%), and total tumor size of ≤1 cm was found in 92 patients (21.9%). Multifocality was present in 149 patients (35.4%), and 96 patients (22.8%) had ETE. We excluded all patients with multiple tumors on the LLNM side when analyzing the relationship between the tumor location and LLNM because we could not determine which tumor caused the LLNM. The numbers of patients by primary tumor location were: upper (166 cases, 40.1%), middle (156 cases, 37.7%), lower (66 cases, 16%), and full (26 cases, 6.3%). Moreover, the numbers of patients by T stage were: T1 (240 cases, 57%), T2 (72 cases, 17.4%), T3 (49 cases, 11.6%), and T4 (60 cases, 14.3%).

The mean number of harvested ipsilateral CLNs in the 421 patients was 4.6 ± 3.5. The mean number of harvested LLNs was 15.4 ± 7.8, of which 4.0 ± 2.8 were confirmed as having metastases. The most frequently involved LLN level was level III with 301 patients (71.5%) with metastases, followed by level IV with 281 patients (66.7%; Table 1).

**TABLE 1:** Clinical characteristics of all included patients with PTC and LLNM, and a comparison between patients with (group A) or without (group B) CLNM.

	All patients	Patients with lateral lymph node metastasis (*N* = 421)		
		With CLNM (group A), *n* = 344 (81.7%)	Without CLNM (group B), *n* = 77 (18.3%)	*p*‐value
Sex				
Male	124 (29.5)	105 (84.7)	19 (15.3)	0.309
Female	297 (70.5)	239 (80.5)	58 (19.5)	
Age (years)	40.5 ± 12.1			
≥55	58 (13.8)	39 (67.2)	19 (32.8)	0.002
<55	363 (86.2)	305 (84.0)	58 (16.0)	
Tumor size	2.0 ± 1.1			
>1	307 (73.0)	265 (86.3)	42 (13.7)	<0.001
≤1	114 (27.0)	79 (69.3)	35 (30.7)	
Total tumor size^a^	2.2 ± 1.3			
>1	311 (73.9)	282 (85.7)	49 (14.3)	<0.001
≤1	92 (26.1)	62 (67.4)	30 (32.6)	
Multifocality				
Yes	149 (35.4)	126 (84.6)	23 (15.4)	0.262
No	272 (64.6)	218 (80.1)	54 (19.9)	
ETE				
Yes	96 (22.8)	80 (83.3)	16 (16.7)	0.64
No	325 (77.2)	264 (81.2)	61 (18.8)	
Nodular goiter				
Yes	236 (56.1)	186 (78.8)	50 (21.2)	0.082
No	185 (43.9)	158 (85.4)	27 (14.6)	
Hashimoto's thyroiditis				
Yes	119 (28.3)	102 (85.7)	17 (14.3)	0.182
No	302 (71.7)	242 (80.1)	60 (19.9)	
Location				
Upper	166 (40.1)	127 (76.5)	39 (23.5)	0.033
Middle	156 (37.7)	131 (82.9)	27 (17.1)	
Lower	66 (16)	61 (92.4)	5 (7.6)	
Full	26 (6.3)	21 (80.8)	5 (19.2)	
T stage				
I	240 (57)	189 (78.8)	51 (21.2)	0.286
II	72 (17.4)	63 (87.5)	9 (12.5)	
III	49 (11.6)	40 (81.7)	9 (18.3)	
IV	60 (14.3)	52 (86.7)	8 (13.3)	
CLN (ipsilateral)	4.6 ± 3.5			
Harvested		5.0 ± 3.5	2.8 ± 2.7	<0.001
Metastatic		3.1 ± 2.5	0	–
LLNM case				
II	220	193 (87.8)	27 (12.2)	0.9
III	301	257 (85.4)	44 (14.6)	
IV	281	242 (86.1)	39 (13.9)	
V	51	44 (86.3)	7 (13.7)	
LLNM number	4.0 ± 2.8	4.4 ± 2.9	2.4 ± 1.9	<0.01
II	0.9 ± 1.2	1.0 ± 1.3	0.5 ± 0.9	<0.01
III	1.5 ± 1.5	1.7 ± 1.6	1.0 ± 1.2	<0.01
IV	1.3 ± 1.4	1.5 ± 1.5	0.8 ± 1.1	<0.01
V	0.2 ± 0.8	0.3 ± 0.9	0.1 ± 0.4	0.19
LLN (H) number	15.4 ± 7.8	15.5 ± 7.9	15.5 ± 7.10	0.63
II	4.5 ± 3.4	4.6 ± 3.6	4.4 ± 3.0	0.68
III	4.6 ± 3.1	4.6 ± 3.2	4.6 ± 3.3	0.91
IV	4.4 ± 3.2	4.5 ± 3.3	4.1 ± 2.7	0.31
V	1.9 ± 2.6	1.9 ± 2.6	2.0 ± 2.8	0.71

Abbreviations: CLN, central lymph node; CLNM, central lymph node metastasis; ETE, extrathyroidal extension; LLN, lateral lymph node; LLNM, lateral lymph node metastasis; LLNM case, the number of patients with LLNM; LLNM number, the number of metastasis lateral lymph node; LLN (H), lateral lymph node (harvested); PTC, papillary thyroid cancer; SD, standard deviation.

^a^ Total tumor size: When the patient has more than one tumor, we calculate the sum of all tumor diameters as total tumor size.

Seventy‐seven patients (18.3%) had LLNM without CLNM. We grouped the patients into two groups, those with (group A) and those without (group B) CLNM, and compared their clinical characteristics. The results indicated that individuals aged ≥55 years (*p* = 0.002), those with primary tumor size or total tumor size <1 cm (*p* < 0.001), and those with the primary tumor in the upper portion of the thyroid (*p* = 0.033) were more likely to have LLNM without CLNM (Table 1). Moreover, the number of LNM in group B was smaller than in group A in levels II, III, and IV (*p* < 0.001), and fewer lymph nodes of level VI were harvested in group B. The groups were similar for sex, ETE, multifocality, HT or goiter nodular, and the number of harvested LLNs.

Multivariable logistic regression analysis was used to analyze the factors after adjusting for the remaining factors: sex, harvested CLN number, and tumor size. The obtained results indicated that the incidence of LLNM without CLNM was higher in patients aged ≥55 years (OR, 2.309; 95% CI, 1.133–4.704; *p* = 0.021) and patients whose primary tumor was in the upper portion of the thyroid (OR, 0.524; 95% CI, 0.295–0.934; *p* = 0.028; Table 2).

**TABLE 2:** Multivariate logistic regression analyses of factors contributing to lateral lymph node metastasis without central lymph node metastasis in patients with PTC.

Variables	Odds ratio	95% CI	*p*‐value*
Age (≥55)	2.309	1.133–4.704	0.021
Primary tumor location (upper/other sites)	0.524	0.295–0.934	0.028

Abbreviations: CI, confidence interval; PTC, papillary thyroid cancer.

* *p*‐value for the independent association between lateral lymph node metastasis without central lymph node metastasis and each factor after adjusting for the remaining factors, sex, harvested central lymph node number, and tumor size.

Up to January 2021, we followed up 348 patients (82.66%) for 48–96 months (mean, 72 months), including 296 of the patients in group A (86.05%) and 61 patients in group B (79.22%). Two patients (one from each group) died of other diseases during the follow‐up, and nine (2.59%) had recurrent disease. CLNM recurrence occurred in two patients in group B (3.28%). The recurrence rate in group A was 2.36% (7/296), three patients in the CLN and four in the LLN. The groups were statistically similar for the recurrence rates (2.36% vs. 3.28%, *p* = 0.654).

## 4 DISCUSSION

PTC has an optimistic prognosis. The results of previous studies have indicated that several factors were likely to affect the patient outcome, and LNM plays a critical role among them. LLND has not been routinely implemented in most countries, including some Asian countries, unless there was conclusive evidence or considerable suspicion of metastasis. However, the CLN dissection has been routinely performed in most patients with PTC in our department and some other hospitals. Previous studies have reported that LLNM was associated with local recurrence and a worse prognosis.^12^, ^13^ LLNM without CLNM occurred in this study in 18.29% of the patients (77/421). If we focus on whether the patients have CLNM without evaluating the LLN, the LLNM of such patients might be missed.

The ultrasonography and enhanced CT were useful in diagnosing lymph node metastases, but there must be some omission for patients. A previous meta‐analysis reported that the accuracy of ultrasonographic detection of LNM was 70%.^11^ Moreover, fine needle aspiration has become the most routinely used tool for preoperative PTC and LNM diagnosing. However, it is not routinely used, particularly not in cN0 patients.

This study's findings indicated that LLNM without CLNM was uncommon, but should not be ignored. The occurrence of LLNM without CLNM could have several mechanisms. First, PTC might send metastases to the LLN along the superior thyroid artery instead of the classic lymphatic drainage system, the standard route of PTC metastases. Our results also indicated that LLNM without CLNM was more common when the primary tumor was in the upper portion of the thyroid. The dissection of the CLN would be meaningless for such patients and sometimes even harmful. Previous studies have reported that the incidence of transient hypocalcemia in patients following total thyroidectomy ranged between 8.3% and 36.5%, compared with 23.2%–51.9% in patients with total thyroidectomy and CLND.^14^, ^15^ Harries et al. reported that cN0 patients with LLNM had a low recurrence rate in the central region, even if CLND was not performed during the initial operation. Consequently, such patients would not benefit from prophylactic CLND.^16^ Therefore, surgeons need to evaluate whether the patients have LLNM without CLNM, as these findings could help determine the necessity of CLND. Second, there might be some false LLNM without CLNM due to missed CLNM. This might be because the pathologist has missed the CLNM or because the surgical removal was incomplete. This could affect the staging and accuracy of follow‐up. Therefore, surgeons should be careful and highly skilled to avoid missing metastasized lymph node.

The rate of LLNM without CLNM was 18.29% (77/421), consistent with findings in previous studies that ranged between 5% and 25%.^17^, ^18^, ^19^ In recent years, many research centers, including the Kuma Hospital in Kobe, Japan, have been actively monitoring patients with papillary thyroid microcarcinoma (PTMC) rather than administering surgical treatment. Active monitoring has been enacted in many patients with PTC, not only in those with PTMC.^20^ Furthermore, most researchers have realized the low malignancy grade of PTC and consider its treatment to be excessive. However, it is worth noting that a small tumor does not mean low malignancy. The findings of our study showed that patients with a tumor of less than 1 cm in size, including primary and total tumor diameters, were more likely to have LLNM without CLNM, consistent with findings reported in previous studies. Based on these analyses, we speculate that more time is required for cancer to metastasize to the CLN than the LLN due to the inertia of the PTC cells. The increasing popularity of ultrasonography has resulted in diagnosing and treating many patients with PTC before cancer has metastasized to the CLN and LLN. Besides, several scholars have recognized that the tumor size does not affect the rate of CLNM.^21^, ^22^ Recently, researchers in Mexico compared the risk stratification between the Kuma criteria and the 2015 ATA guidelines. They reported that some patients who met the Kuma criteria required more aggressive management than active monitoring.^23^ However, we disagree with the opinion that PTMC has been over‐treated. Therefore, the development of a treatment strategy for PTMC requires more specific standards and strict evaluation, not to miss some cases because of the assumed good prognosis of PTMC. Such missed diagnoses might reduce their survival time and quality of life.

Several previous studies have reported that LLNM without CLNM was associated with factors such as multifocality^24^ and ETE,^8^, ^25^ in addition to the upper portion of the thyroid and PTMC. However, this study found no such association for these factors, possibly due to selection bias introduced by the inclusion and exclusion criteria. Most researchers have speculated that the PTC foci originate from intra‐thyroidal metastasis of the same clonal cell population or independent initiation of multiple tumors.^26^ Different clonal origins might lead to different biological characteristics. Moreover, considerable controversy surrounds the choice of a surgical procedure for patients with multiple PTC foci.^27^, ^28^ Therefore, more studies are required to confirm the relationship between multifocality and LLNM without CLNM.

This study had several limitations. First, there is an undeniable bias in the accuracy of the results in all retrospective studies, even in our, which study included more patients than most previous retrospective studies on the topic. Second, some of our patients underwent lobectomy with ipsilateral CLND and LLND; therefore, contralateral lobe occult cancer or occult CLNM may have been missed. Third, LLND was not routinely performed in all patients with PTC; therefore, some LLNM might have been missed, affecting our results. Therefore, a future multi‐center prospective study with a large sample could be used to analyze the risk factors of LLNM without CLNM in patients with PTC.

In conclusion, we suggest that patients with no CLNM but lateral cervical lymph node metastasis should not be referred to as skip metastasis but rather as LLNM without CLNM. Besides, this study results indicated that LLNM without CLNM should be considered before surgery, especially in patients older than 55 years and when the tumor is in the upper portion of the thyroid. Finally, preoperative evaluation of patients with PTC should be comprehensive and performed with caution.

## AUTHOR CONTRIBUTIONS

**Liang Shao:** Investigation (lead); project administration (equal); writing – original draft (lead); writing – review and editing (lead). **Zhihong Wang:** Investigation (equal); software (equal); validation (equal). **Wenwu Dong:** Formal analysis (equal); investigation (equal); methodology (equal); visualization (equal). **Wei Sun:** Data curation (equal); formal analysis (equal); investigation (equal); software (equal). **Hao Zhang:** Conceptualization (lead); project administration (lead); supervision (lead).

## FUNDING INFORMATION

This work was supported by the National Natural Science Foundation of China (grant number 81902726).

## CONFLICT OF INTEREST STATEMENT

The authors declare no conflicts of interest.

## Data Availability

I confirm that I have included a citation for available data in my references section, unless my article type is exempt
